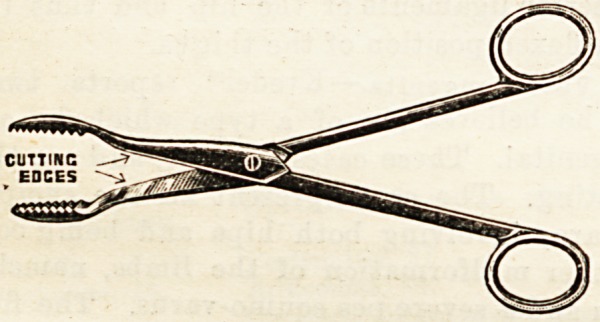# New Appliances and Things Medical

**Published:** 1898-07-16

**Authors:** 


### NEW APPLIANCES AND THINGS MEDICAL.

[We shall be glad to receive, at our Office, 28 & 29, Southampton Street, Strand, London, W.C., from the manufacturers, specimens of all new preparations and appliances which may be brought out from time to time.]

### COMBINATION SURGICAL SCISSORS AND DRESSING FORCEPS.

Devised by Mr. H. de Paiva B. Veale, late House Surgeon Leeds General Infirmary.

(Messrs. Reynolds and Branson, Limited, Leeds)

Combination instruments are not always very successful, and it would be easy to point to many examples in which the instrument maker has thrown away substantial utility in his endeavour to make one instrument serve for many purposes. This, however, is not the case in the useful little combination of scissors and forceps which has been contrived by Mr. Veale. Everyone must often have wished that his dressing forceps would cut or that his scissors could be used for removing dressings, and here is just the combination wanted. The points are the points of dressing forceps, but the blades are those of a pair of scissors. It may also be used as an emergency "clip" or as a sinus dilator, a rib having been left on the unsharpened edges so as to render it available for the latter purpose. In the sample sent to us the blades are riveted, but we are informed that it can be obtained with detachable blades. This we look upon as essential in an instrument which may require to be thoroughly cleansed many times in a morning's round. The fixed blades must in such a case be a very objectionable arrangement. Every dodge which lessens the bulk of the pocket case is a distinct advantage to the general practitioner, while to house surgeons, dressers, and nurses the utility of this little contrivance for rough purposes is obvious.

### NATURAL WHOLE-WHEAT BREAD.

(Callard, Stewart, and Watt, Limited, 176, Piccadilly, London, W.)

This new bread, which is known as the N. A. P. brand, is a carefully and skilfully prepared product, containing a large percentage of albuminoids and phosphates. As a food it must be regarded as greatly superior to the ordinary breads made from refined flour, and from our experience it appears readily digested and assimilated. For growing children and those undergoing severe physical exercise, it is an economical and satisfactory substitute for the more expensive flesh formers, and in children with a tendency to rickets it has the additional advantage of containing a high percentage of phosphates. As compared to ordinary bread the N. A. P. brand has many advantages, and as far as we can judge there is no condition that contra-indicates its substitution for it.

### PREPARED COCOA.

(James Epps, Limited, Holland Street, Blackfriars, London, S E)

This old-established and well-known cocoa has been submitted to our notice. It still has so large a following of admirers, especially among children, that in spite of many of its largely-advertised rivals it still holds a pre-eminent position among them. Unlike many other varieties the natural oil of the nut has not been removed, but it is rendered easy of admixture with water or milk by a combination with arrowroot and sugar. Beyond these two ingredients we can detect no addition or impurities. It is a wholesome and readily-digested food, and from long experience we can recommend it for children from two years upwards. They prefer it to the pure extracts and soluble cocoas, and as far as we can detect there is no contra-indication for its use.

## Figures and Tables

**Figure f1:**